# Radiochemotherapy in Pancreatic Cancer

**DOI:** 10.3390/curroncol31060250

**Published:** 2024-06-06

**Authors:** Małgorzata Domagała-Haduch, Anita Gorzelak-Magiera, Łukasz Michalecki, Iwona Gisterek-Grocholska

**Affiliations:** Department of Oncology and Radiotherapy, Medical University of Silesia, 40-514 Katowice, Poland; anitagor@op.pl (A.G.-M.); lukaszmichalecki@gmail.com (Ł.M.); igisterek@sum.edu.pl (I.G.-G.)

**Keywords:** radiochemotherapy, unresectable pancreatic cancer, resectable pancreatic cancer, borderline resectable pancreatic cancer, chemotherapy

## Abstract

Despite the advancements made in oncology in recent years, the treatment of pancreatic cancer remains a challenge. Five-year survival rates for this cancer do not exceed 10%. Among the reasons contributing to poor treatment outcomes are the oligosymptomatic course of the tumor, diagnostic difficulties due to the anatomical location of the organ, and the unique biological features of pancreatic cancer. The mainstay of treatment for resectable cancer is surgery and adjuvant chemotherapy. For unresectable and metastatic cancers, chemotherapy remains the primary method of treatment. At the same time, for about thirty years, there have been attempts to improve treatment outcomes by using radiotherapy combined with systemic treatment. Unlike chemotherapy, radiotherapy has no established place in the treatment of pancreatic cancer. This paper addresses the topic of radiotherapy in pancreatic cancer as a valuable method that can improve treatment outcomes alongside chemotherapy.

## 1. Introduction

Pancreatic adenocarcinoma is one of the most poorly treatable cancers. Five-year survival rates for this cancer do not exceed 10% [1]. With an annual death toll of about 128,000, the cancer ranks as the fourth most common cause of cancer death [2].

It is estimated that pancreatic cancer will become the second cause of cancer deaths in 2030 [3].

The only method to achieve long-term survival is primary surgery with subsequent follow-up chemotherapy. Unfortunately, due to the oligosymptomatic progression of the disease, resectable cancer is diagnosed in 10–20% of patients. About 40% of patients are diagnosed with stage IV disease, while 40–50% of patients have locoregional, inoperable pancreatic cancer at the time of diagnosis [4].

The most important criterion for unresectability of pancreatic cancer is infiltration of the surrounding arterial and venous vessels. Based on the type of vessels involved and the extent of infiltration, pancreatic cancer has been divided into resectable, borderline resectable, and unresectable [5,6].

This article discusses the role of radiochemotherapy in the treatment of pancreatic cancer according to the stage of the tumor.

## 2. Resectable Pancreatic Cancer

Resectable pancreatic cancer is defined as a lesion that does not infiltrate the vessels of the visceral trunk, superior mesenteric artery, or common hepatic artery. As regards the venous vessels, the lesion must not infiltrate the superior mesenteric vein or portal vein (involvement of <180° of these vessels without obliteration of their wall is allowed) [6].

In resectable tumors, the standard procedure is pancreatoduodenectomy. Depending on anatomopathological considerations, this can be a pancreatoduodenectomy, Whipple method, involving resection of the head of the pancreas with the duodenum and part of the stomach and the gallbladder with the common bile duct followed by reconstruction of the digestive system involving anastomosis of the pancreatic body with the intestine or stomach and anastomosis of the common hepatic duct and stomach with the jejunum.

In the case of reconstruction with preservation of the entire stomach and the initial portion of the duodenal pad, the operation is defined as pancreatoduodenectomy with sparing of the pylorus (Traverso method).

In tumors located in the distal part of the pancreas, the preferred procedure is peripancreatic resection by a block progressive resection of the left part of the pancreas with a resection of the spleen and the lymphatic system of the retroperitoneal space.

Chemotherapy should be considered in every patient after surgery because of its significant impact on improving patient outcomes. Unfortunately, up to half of patients may not receive adjuvant treatment, mainly due to postoperative complications and clinical deterioration, as well as early recurrence of the cancer [7,8].

The preferred regimen for adjuvant chemotherapy is the modified FOLFIRINOX regimen (5-fluorouracil by continuous infusion, oxaliplatin, irinotecan) due to the best results of such therapy in phase III trials. In the registration study, the use of mFOLFIRINOX chemotherapy resulted in a median overall survival (OS) of 54.4 months. In the control group, which received gemcitabine chemotherapy, the median OS was 35 months (*p* = 0.003) [9].

With contraindications to three-drug chemotherapy, two-drug chemotherapy with capecitabine plus gemcitabine is possible. In the ESPAC-4 trial, such treatment resulted in a 28-month median overall survival time. In the gemcitabine-treated control group, the median OS was 25.5 months (*p* = 0.032) [10].

Currently, nab-paclitaxel with gemcitabine is not recommended as adjuvant therapy. The APACT trial showed an improvement in overall survival time compared to adjuvant therapy with gemcitabine (mOS in the study group was 41.8 months vs. 37.7 months in the control group, *p* = 0.091), but the trial did not show an improvement in treatment outcome with respect to the primary endpoint of disease-free survival (DFS) [11].

Complementary monotherapy based on gemcitabine or 5-fluorouracil is allowed in special situations [6].

In operable cancer, neoadjuvant chemotherapy is a subject of controversy. U.S. recommendations allow the use of such treatment in the presence of risk factors such as significantly a elevated Ca19.9 marker, large tumor mass, regional lymph node involvement, rapidly progressive weight loss, or severe pain [6].

In contrast, the recently published NORPACT-1 trial showed that, in resectable tumors, neoadjuvant treatment with the FOLFIRINOX regimen worsens the prognosis compared to patients undergoing primary surgery. In the intent-to-treat (ITT) population, the median survival time in the primary surgery group was 38.5 months, while in the perioperative therapy group it was 25.1 months (*p* = 0.05). A difference was also demonstrated in the 18-month survival rate—73% vs. 60% (*p* = 0.032) [12].

In the treatment of operable cancer, radiotherapy and radiochemotherapy are routinely not used. In the ESPAC-1 trial, adding a split-course radiotherapy of 2 Gy for five fractions per week for 2 weeks, repeated after 2 weeks (total 40 Gy), to adjuvant chemotherapy with 5-fluorouracil did not translate into a prolonged overall survival rate. Moreover, the use of radiochemotherapy before systemic treatment resulted in a shorter median OS in the study subgroup: 13.9 months in the chemoradiotherapy group, 19.9 months in the group treated with chemotherapy with the addition of radiochemotherapy, and 21.6 months in the group treated with chemotherapy alone. That study also showed that even patients with positive postoperative margins did not benefit from postoperative radiochemotherapy [13].

Adjuvant radiochemotherapy has also been the subject of meta-analyses.

A 2005 meta-analysis involving five clinical trials with 875 patients examining the effect of adjuvant treatment showed that radiochemotherapy as adjuvant treatment had no effect on reducing the risk of death in patients. The most commonly used was a split course of RT 2 Gy for five fractions per week for 2 weeks, repeated after 2 weeks (total 40 Gy) [14].

A meta-analysis by Ren et al. [15] analyzed data from 15 prospective randomized trials (4099 patients). The studies used different cytostatics (cisplatin, gemcitabine, mitomycin, cisplatin) and different doses of radiotherapy (20–50.4 Gy). The results of the meta-analysis showed that adjuvant radiochemotherapy did not improve parameters such as disease-free survival (DFS), overall survival (OS), and two-year survival rates compared to surgery alone; however, complementary chemotherapy benefited all three parameters.

Another meta-analysis published a year later in the journal The Lancet analyzed data from 10 studies (3033 patients). It showed that chemoradiotherapy with gemcitabine or 5-fluorouracil and a radiation dose of 20–40 Gy yielded worse parameters with regard to overall survival compared to chemotherapy with either drug; the combination treatment was also associated with higher toxicity [16].

Currently, there are no results on adjuvant chemoradiotherapy based on the FOLFIRINOX treatment scheme.

A meta-analysis analyzing the benefit of adding radiotherapy to neoadjuvant chemotherapy according to the FOLFIRINOX regimen showed that the radiochemotherapy group achieved a higher R0 resection rate (97% vs. 88%), but this did not translate into prolonged overall survival [17]. 

## 3. Borderline Resectable Pancreatic Cancer

Borderline resectable pancreatic cancer is considered to be tumor in contact with <180° of the superior mesenteric artery and visceral trunk or in contact with >180° of the portal vein and superior mesenteric vein with possible resection. 

Borderline resectable pancreatic cancer is a tumor in which primary surgery is not recommended because of the very high risk of not achieving surgical completion. When non-radical resection is performed, the overall survival time achieved is comparable to that of patients who do not undergo surgery [18].

Based on the above, it is recommended that, for this stage of pancreatic cancer, treatment should begin with chemotherapy and/or radiochemotherapy aimed at conversion to operable cancer [6].

Currently, the treatment of patients with borderline resectable pancreatic cancer is mainly based on the use of chemotherapy based on the FOLFIRINOX regimen or its modified version (mFOLFIRINOX).

The use of FOLFIRINOX chemotherapy in borderline resectable pancreatic cancer enables R0 resection in nearly 70% of patients; among surgical patients, R0 margins are achieved by 84% of patients, and the median OS reaches 34 months [19]. 

The PREOPANC trial, which enrolled 248 patients with resectable and potentially resectable pancreatic cancer in the experimental arm, used primary surgery supplemented with gemcitabine-based adjuvant chemotherapy in the control group. The study group received neoadjuvant chemoradiotherapy with gemcitabine, followed by surgery and postoperative gemcitabine-based chemotherapy. 

The use of radiochemotherapy resulted in a five-year survival rate of 20.5%, while the control group had an OS rate of 6.5% (*p* = 0.025). Improved OS was noted in both subgroups—both in patients with resectable and potentially resectable cancer. An R0 margin was achieved in 41% of patients previously treated with combination therapy; in the surgically treated group, an R0 margin was achieved in 28% of patients [20].

It should be noted that that study conducted irradiation with higher fractional doses—2.4 Gy, administering 36 Gy in 15 fractions.

A phase II study published in 2018 analyzed the possibility of improving the treatment of patients with borderline resectable pancreatic cancer by adding chemoradiotherapy to eight cycles of induction chemotherapy with the FOLFIRINOX regimen. Patients who achieved conversion to operable cancer after systemic treatment underwent proton therapy with a dose of 25 Gy in five fractions with the addition of capecitabine followed by radical surgery. Patients who did not achieve resectability after FOLFIRINOX chemotherapy were qualified for photon beam radiotherapy using the IMRT technique (58.5 Gy in 28 fractions) with concurrent chemotherapy with capecitabine or 5-fluorouracil. 

Radical surgery was performed in 66% of patients, and 97% of them achieved R0 margins. The experimental group had a median PFS of 14.7 months, while the surgery group had a median PFS of 48.6 months. The median OS for the entire experimental group was 37.7 months. In the operated group, the two-year OS rate was 72 [21].

In the ALLIANCE trial involving 126 patients with borderline resectable pancreatic cancer, eight cycles of mFOLFIRINOX chemotherapy were administered in the control group and seven cycles of mFOLFIRINOX chemotherapy with stereotactic radiotherapy (33–40 Gy in five fractions) or IGRT radiotherapy (25 Gy in five fractions) in the study group. Patients who underwent surgery received four cycles of the FOLFOX 6 regimen in complementary chemotherapy. 

That study showed the superiority of stand-alone chemotherapy in almost all aspects: in the first evaluation, R0 resection was achieved in 57% of patients treated with chemotherapy; in the experimental group, R0 resections were achieved in 33% of the patients. An OS of 18 months was achieved in 67% of patients in the control group and 47% in the experimental group. Thus, the results of the ALLIANCE trial showed that combination treatment is not superior to chemotherapy alone [22]. 

In the PREOPANC-2 trial, recently published in an abstract form, neoadjuvant chemotherapy according to the FOLFIRINOX regimen (eight cycles) or gemcitabine-based radiochemotherapy (36 Gy in 15 fractions) was administered in a group of patients with resectable and potentially resectable pancreatic cancer (patients undergoing tumor resection received four courses of supplemental gemcitabine chemotherapy after surgery) The primary endpoint was overall survival time. Radical surgery was performed in 77% of patients undergoing FOLFIRINOX chemotherapy and in 75% of patients treated with radiochemotherapy. The median OS in the group treated with chemotherapy alone was 21.9 months, while, in the combination treatment group, it was 21.3 months (*p* = 0.28). The results of that study show that, for patients with borderline resectable pancreatic cancer and with contraindications to three-drug chemotherapy, the use of gemcitabine-based radiochemotherapy may be a valuable treatment option [23].

## 4. Unresectable Pancreatic Cancer without Distant Metastases

Unresectable pancreatic cancer is considered to be tumor contact with the superior mesenteric artery or coeliac axis >180 or tumor contact with the first jejunal superior mesenteric artery branch. It also includes the case of an unreconstructible superior mesenteric vein or portal vein due to tumor involvement or occlusion or contact with the most proximal draining jejunal branch into the superior mesenteric vein.

Unfortunately, 80% of pancreatic cancers are diagnosed at an advanced stage, of which 50% are patients diagnosed at the metastatic stage, and 30% are patients with unresectable locally advanced pancreatic cancer (LAPC). The overall survival time of patients with definitively unresectable pancreatic cancer compared to patients in the disseminated stage is significantly longer (10–12 months vs. 5–6 months), which translates into the need for different types of therapy in these two groups of patients [24,25,26,27].

For years, chemotherapy has been the standard treatment for advanced pancreatic cancer. The use of the FOLFIRINOX regimen in initially unresectable patients allows conversion to resectable cancer in about 20% of cases. However, it should be noted that, in the cited article, a condition in which the tumor invades the celiac trunk in any way is defined as a non-resectable process. According to the NCCN definition, this type of cancer is classified as borderline resectable, so patients with potentially resectable cancer could participate in that study [28,29]. 

In 1981, the first study of 194 patients with locally advanced unresectable pancreatic cancer was published, in which patients were divided into three subgroups. The first arm received 60 Gy of radiotherapy alone; the second arm received 40 Gy of radiotherapy with 5-fluorouracil; and the third arm received 60 Gy of radiotherapy with 5-fluorouracil. The chemoradiotherapy arms received maintenance treatment with 5-fluorouracil for 2 years or until disease progression. There was a statistically significant benefit in terms of overall survival time in the group of patients receiving the combination treatment, compared to those treated with radiation therapy alone. The overall survival times were 5.3 vs. 9.7 vs. 9.3 months, respectively. In contrast, there was no statistical significance between the combination treatment groups. It should be emphasized that that study was historical because the radiotherapy techniques were completely different from those used today [30].

In locoregionally advanced, unresectable pancreatic cancer, radiotherapy is one of the methods to improve local control of the disease and result in an increased rate of radical resection (R0), although published studies to date have not conclusively resolved its efficacy or optimal protocol for use [6]. In a prospective randomized study by Shinchi et al. [31], 16 patients received chemoradiotherapy (50.4 Gy in 28 fractions with a concomitant infusion of fluorouracil 200 mg/m^2^/day, followed by maintenance fluorouracil treatment), while a control group of 15 patients received neither chemotherapy nor radiotherapy. The survival time for patients undergoing the combination treatment was significantly longer (OS 13.2 vs. 6.4 months). There was also a significant improvement in the quality of life of the treated patients.

In contrast, a study by Cohen et al. [32] that included 104 patients compared the results of treatment with 59.4 Gy of radiotherapy alone in 33 fractions with chemoradiotherapy using fluorouracil 1000 mg/m^2^ administered on days 2–5 and 28–31 of radiotherapy and mitomycin 10 mg/m^2^ on day 1 and showed no benefit from the combination treatment, observing a significantly higher toxicity [32]. 

The benefit of concomitant radiotherapy (54 Gy/30 fr) with chemotherapy (5-fluorouracil 350 mg/m^2^ bolus on days 1–3 and 36–38) compared to chemotherapy alone with 5-fluorouracil 600 mg/m^2^ bolus on days 1, 8, 29, 36; streptozocin 1 g/m^2^; and mitomycin 10 mg/m^2^ on day 1 every 8 weeks was obtained in the GITSG trial in which the 1-year survival was longer in patients treated in a combination fashion (41% vs. 19% *p* < 0.02) [33].

Chauffert et al. [34], in a group of 119 patients, showed shorter survival (13 months vs. 8.6 months *p* = 0.03) in the chemoradiotherapy arm (60 Gy in 30 fractions, infusion of 5-fluorouracil at a dose of 300 mg/m^2^/day on days 1–5, cisplatin 20 mg/m^2^/day on days 1–5 in weeks 1 and 5). In the stand-alone chemotherapy arm, gemcitabine at a dose of 1000 mg/m^2^ administered every 7 days was used. The maintenance therapy was gemcitabine at a dose of 1000 mg/m^2^.

A phase II clinical trial involving 25 patients analyzed the effect of chemotherapy preceding radiochemotherapy. The induction treatment included cisplatin with gemcitabine followed by radiotherapy at a dose of 50.4 Gy in 28 fractions with capecitabine and showed prolonged overall survival in the combination treatment arm (17 months vs. 13.5 months for all including patients) [35]. In a similar study by Huguet et al. [36] involving 181 patients, it was shown that, in the radiochemotherapy-treated group (54 Gy/30 fractions with 5-fluorouracil 250 mg/m^2^/day for 7 days a week), after at least 3 months of induction chemotherapy, the overall survival time was 15 months, compared to 11.7 months in the chemotherapy-treated group (*p* = 0.0009). A benefit was also shown in terms of time free from disease progression (10.8 vs. 7.4 months, *p* = 0.005).

Phase III of the LAP07 trial, which enrolled 449 patients, compared the use of gemcitabine 1000 mg/m^2^ and gemcitabine 1000 mg/m^2^ with erlotinib 100 mg/m^2^. In a subsequent randomization, patients without cancer progression after 4 months of treatment were eligible for continued chemotherapy or chemoradiotherapy (54 Gy in 30 fractions with capecitabine 800 mg/m^2^ twice daily). There was no significant advantage of combination treatment (15.2 vs. 16.4 months) [37].

Despite the disparity in study results, induction chemotherapy for 3–4 months followed by chemoradiotherapy remains a treatment option for local disease control. Among the numerous chemotherapeutic agents studied for combination treatment, fluoropyrimidines and gemcitabine were the most common choices. In the phase II SCALOP trial, gemcitabine 1000 mg/m^2^ was administered for 12 weeks at a dose of 1, 8, 15 every 28 days and capecitabine 830 mg/m^2^ twice daily on days 1–21 with a 7-day break, and chemoradiotherapy (50.4 Gy in 28 fractions in combination with gemcitabine 300 mg/m^2^ every week or capecitabine 830 mg/m^2^ for 5 days per week) was initiated after a randomization and continuation of chemotherapy at the same doses as were used during induction [38].

According to Loehrer et al. [39], gemcitabine-based radiochemotherapy leads to increased treatment toxicity—the rate of serious adverse events (G4) in the combination treatment was significantly higher than in the gemcitabine-alone arm (41% vs. 9%). The percentage of toxicity in grade 2 and 3 was similar in both arms (77% vs. 79%). Moreover, it showed no statistical difference in quality of life between both groups. 

## 5. Summary and Discussion

Due to the biology of the tumor, chemotherapy is used at any stage of pancreatic cancer if the patient’s general condition allows such therapy. Despite many years of research, radiochemotherapy has not found an established place in the treatment of this type of cancer. In resectable tumors, radiochemotherapy using regimens based on gemcitabine or 5-fluorouracil is not routinely used, although the NCCN recommendations allow such treatment in certain circumstances [6]. In the opinion of the authors of this review, such a procedure may be considered in patients who did not consent or are not eligible for triple therapy. If the tumor has the R1 circumferential resection margin (CRM), radiochemotherapy should be preceded by systemic treatment.

For borderline resectable pancreatic cancer, the advisability of radiochemotherapy is controversial. Although, in a study by Katz et al. [22], the combination of radiotherapy with induction chemotherapy according to the FOLFIRINOX regimen translated into shorter overall survival and lower R0 resection rates, it is noteworthy that that study did not use uniform regimens or irradiation techniques. With such a variety of bioequivalent doses, the question arises as to whether improved outcomes would have been achieved if the modern high-dose bioequivalent radiotherapy technique of SRBT had been applied to all patients. In addition, in the study arm, significantly more patients had reduced doses of induction chemotherapy with mFOLFIRINOX (75% vs. 60%), which could also have translated into the result. 

In the PREOPANC trial, the use of gemcitabine chemotherapy with subsequent radiation therapy with surgery and adjuvant systemic treatment with gemcitabine resulted in an improved OS in patients with resectable and borderline resectable tumors. It is worth noting the resectability criteria adopted by that study’s authors—for borderline resectable tumors, infiltration of the visceral trunk or superior mesenteric artery could not exceed 90° of the vessel’s circumference. For the NCCN criteria for borderline resectable cancer, infiltration of these vessels must not exceed 180° of the circumference. That study showed that a lower tumor resectability rate is achieved with radiochemotherapy (61% vs. 72%), with the differences possibly due to tumor progression during neoadjuvant treatment. According to that study’s authors, patients with progression during neoadjuvant therapy may have a more aggressive course of pancreatic cancer and would experience rapid cancer recurrence if primary surgery were performed. 

In the PROPANC trial, the benefit of postoperative radiochemotherapy was seen in all subgroups, regardless of patient age, lesion resectability, or initial Ca19.9 antigen levels However, a limitation of that study of resectability parameters is the type of treatment used—currently, the three-drug regimen FOLFIRONOX is used for induction as well as adjuvant treatment, rather than gemcitabine, which has a lower efficacy [20].

The PREOPANC 2 trial showed that perioperative chemotherapy with the FOLFIRINOX regimen did not improve the overall survival time compared to radiochemotherapy with gemcitabine in a group of patients with resectable and borderline resectable pancreatic cancer (the treatment regimen was the same as in the PREOPANC trial). These results suggest that, for patients with RBPC and BRPC and contraindications to three-drug chemotherapy, radiochemotherapy with gemcitabine is a reasonable solution [23].

Promising conclusions were published by Murphy et al. [21] showing in patients with BRPC that the combination of FOLFIRINOX chemotherapy and chemoradiotherapy led to resectability in 66% of patients, and the R0 resection rate was 97%. The limitations of this experiment are the number of patients—only 48—and the lack of information on overall survival. 

In the case of inoperable, locoregionally advanced pancreatic cancer, the use of FOLFIRINOX chemotherapy allows for resectability in 20–30% of patients. However, it is worth noting the definitions of unresectable cancer adopted by the authors—in some studies, unresectable cancer is defined as a tumor infiltrating the celiac trunk in any way; in the NCCN definition, such an infiltration should cover more than half of the vessel circumference or contact of the tumor with the celiac trunk should coexist with invasion of the aorta [28,29].

In inoperable, locoregionally advanced pancreatic cancer, radiochemotherapy positively affects the patients’ quality of life and improves their overall survival (OS) compared to symptomatic treatment. However, the results of studies comparing the effects of radiochemotherapy versus chemotherapy alone are conflicting. Based on an analysis of the reports, the optimal approach is to use induction chemotherapy with subsequent radiochemotherapy. It is estimated that, at the time of an LAPC diagnosis, up to 30% of patients may have small distant metastases that are invisible on imaging studies. 

Despite numerous studies and improvement of the techniques used in oncology, the use of radiotherapy in unresectable pancreatic cancer allows for local control in only 50% of patients, reduction of cancer advancement in 1/3 of them, and a complete pathomorphological response to treatment in a marginal percentage of patients. More than 33% of patients who were resectable after the induction treatment achieved a median survival time similar to that achieved in the originally resectable patients, i.e., 20.5 months [40,41,42].

Independent radiotherapy in the treatment of locally advanced disease is used extremely rarely, and irradiation as the only form of treatment can only be considered in patients who do not qualify for systemic treatment [6].

## 6. Future Directions

Due to the poor prognosis of pancreatic adenocarcinomas, numerous studies are being conducted to improve treatment outcomes. In addition to the use of modern radiotherapy techniques, substances are being tested that are intended to sensitize the tumor to the effects of ionizing radiation. An example is hafnium oxide nanoparticles (NBTXR3) administered directly into the tumor. Studies are also underway to determine the effectiveness and toxicity of combining induction chemoradiotherapy with immunotherapy [43].

Another direction is to search for predictive factors allowing the selection of groups of patients who may benefit from treatment intensification. In the SCALOP study, a Ca19.9 concentration <46 IU mL measured after the completion of induction chemotherapy was indicated as a predictor of benefit from chemoradiotherapy [38].

Some hopes are also placed on free circulating DND (ctDNA). The level of ctDNA is lower in patients without tumor metastasis, and this test also allows for the detection of mutations associated with a higher potential for distant spread—a KRAS mutation was detected in 58.9% of patients with disseminated disease and only in 18.2% of patients with locoregionally advanced disease [44,45].

Another promising molecular prognostic factor that may in the future help select patients with slow local progression who could benefit the most from intensified treatment is SMAD4/DPC4—proteins activated by transforming growth factor beta (TGF beta). In an analysis of 641 patients operated on for pancreatic cancer, inactivation of the DPC4 gene was associated with a significantly higher risk of cancer recurrence. In the future, determining the DPC4 gene status may help distinguish a group of patients requiring increased posttreatment follow-up and perhaps more intensive postoperative treatment [46].

Neoadjuvant and induction radiochemotherapy is the subject of many ongoing prospective studies. One of them is a phase II study carried out by the authors of this publication—in that study, we focus on increasing the resectability rate in patients with unresectable and borderline resectable pancreatic cancer by using stereotactic radiotherapy with a dose of 40 Gy in five fractions to the area of infiltrated blood vessels and 30–35 Gy in five fractions on the remaining part of the tumor with simultaneous chemotherapy with capecitabine, followed by chemotherapy based on the mFOLFIRINOX regimen.

## Data Availability

The data presented in this study is available on request from the corresponding author.
